# Xenodiagnosis to evaluate the infectiousness of humans to sandflies in an area endemic for visceral leishmaniasis in Bihar, India: a transmission-dynamics study

**DOI:** 10.1016/S2666-5247(20)30166-X

**Published:** 2021-01

**Authors:** Om Prakash Singh, Puja Tiwary, Anurag Kumar Kushwaha, Shakti Kumar Singh, Dhiraj Kumar Singh, Phillip Lawyer, Edgar Rowton, Rahul Chaubey, Abhishek Kumar Singh, Tulika Kumari Rai, Michael P Fay, Jaya Chakravarty, David Sacks, Shyam Sundar

**Affiliations:** a Department of Medicine, Institute of Medical Sciences, Banaras Hindu University, Varanasi, Uttar Pradesh, India; b Department of Biochemistry, Institute of Science, Banaras Hindu University, Varanasi, Uttar Pradesh, India; c Department of Molecular Biology, Laboratory for Molecular Infection Medicine Sweden, Umea University, Umea, Sweden; d Kala-Azar Medical Research Center, Muzaffarpur, Bihar, India; e Ministry of Environment, Forest and Climate Change, New Delhi, India; f Department of Zoology, Rameshwar College, Babasaheb Bhimrao Ambedkar Bihar University, Muzaffarpur, Bihar, India; g Laboratory of Parasitic Diseases, National Institute of Allergy and Infectious Diseases, National Institutes of Health, Bethesda, MD, USA; h Biostatistics Research Branch, National Institute of Allergy and Infectious Diseases, National Institutes of Health, Bethesda, MD, USA; i Division of Entomology, Walter Reed Army Institute of Research, Silver Spring, MD, USA

## Abstract

**Background:**

Visceral leishmaniasis, also known on the Indian subcontinent as kala-azar, is a fatal form of leishmaniasis caused by the protozoan parasite *Leishmania donovani* and transmitted by the bites of the vector sandfly *Phlebotomus argentipes*. To achieve and sustain elimination of visceral leishmaniasis, the transmission potential of individuals exposed to *L donovani* from across the infection spectrum needs to be elucidated. The aim of this study was to evaluate the relative infectiousness to the sandfly vector of patients with visceral leishmaniasis or post-kala-azar dermal leishmaniasis, before and after treatment, and individuals with asymptomatic infection.

**Methods:**

In this prospective xenodiagnosis study done in Muzaffarpur district of Bihar, India, we included patients with clinically confirmed active visceral leishmaniasis or post-kala-azar dermal leishmaniasis who presented to the Kala-Azar Medical Research Center. These participants received treatment for *L donovani* infection. We also included asymptomatic individuals identified through a serosurvey of 17 254 people living in 26 high-transmission clusters. Eligible participants were aged 12–64 years, were HIV negative, and had clinically or serologically confirmed *L donovani* infection. During xenodiagnosis, the forearms or lower legs of participants were exposed to 30–35 female *P argentipes* sandflies for 30 min. Blood-engorged flies were held in an environmental cabinet at 28°C and 85% humidity for 60–72 h, after which flies were dissected and evaluated for *L donovani* infection by microscopy and quantitative PCR (qPCR). The primary endpoint was the proportion of participants with visceral leishmaniasis or post-kala-azar dermal leishmaniasis, before and after treatment, as well as asymptomatic individuals, who were infectious to sandflies, with a participant considered infectious if promastigotes were observed in one or more individual flies by microscopy, or if one or more of the pools of flies tested positive by qPCR.

**Findings:**

Between July 12, 2016, and March 19, 2019, we recruited 287 individuals, including 77 with active visceral leishmaniasis, 26 with post-kala-azar dermal leishmaniasis, and 184 with asymptomatic infection. Of the patients with active visceral leishmaniasis, 42 (55%) were deemed infectious to sandflies by microscopy and 60 (78%) by qPCR before treatment. No patient with visceral leishmaniasis was found to be infectious by microscopy at 30 days after treatment, although six (8%) were still positive by qPCR. Before treatment, 11 (42%) of 26 patients with post-kala-azar dermal leishmaniasis were deemed infectious to sandflies by microscopy and 23 (88%) by qPCR. Of 23 patients who were available for xenodiagnosis after treatment, one remained infectious to flies by qPCR on the pooled flies, but none remained positive by microscopy. None of the 184 asymptomatic participants were infectious to sandflies.

**Interpretation:**

These findings confirm that patients with active visceral leishmaniasis and patients with post-kala-azar dermal leishmaniasis can transmit *L donovani* to the sandfly vector and suggest that early diagnosis and treatment could effectively remove these individuals as infection reservoirs. An important role for asymptomatic individuals in the maintenance of the transmission cycle is not supported by these data.

**Funding:**

Bill & Melinda Gates Foundation.

## Introduction

Visceral leishmaniasis, also known as kala-azar in the Indian subcontinent, is a deadly tropical disease caused by parasites belonging to the *Leishmania donovani* complex and transmitted by the bites of phlebotomine sandflies.^1^ The disease is endemic in more than 60 countries, with an estimated 50 000–90 000 new cases of visceral leishmaniasis occurring worldwide each year.^2^ The majority of cases occur in six countries: Bangladesh, Brazil, India, Ethiopia, Sudan, and South Sudan.^3^ In 2005, India, Nepal, and Bangladesh agreed to eliminate visceral leishmaniasis as a public health problem by 2015, a deadline that was later reset to 2020.^4^ This target was empirically defined as a reduction of annual visceral leishmaniasis incidence to less than one case per 10 000 population at the health district or subdistrict level.^5^ *Phlebotomus argentipes* is the only proven vector involved in the transmission of visceral leishmaniasis in the Indian subcontinent.^6^ The strategy of the ongoing visceral leishmaniasis elimination programme is early detection and complete treatment of clinical cases and systematic indoor residual spraying.^7^, ^8^ As the three countries get closer to the low-incidence target, new challenges arise, with the main questions being whether the elimination of all *L donovani* transmission from these regions is technically feasible, and what is the best strategy to sustain the elimination targets once achieved.

**Research in context**

**Evidence before this study**

Due to the morbidity associated with visceral leishmaniasis disease (also known as kala-azar in the Indian subcontinent), much research is devoted to preventing transmission of the causative parasite *Leishmania donovani*. Understanding the dynamics and epidemiology of anthroponotic transmission of *L donovani* is important for the development of control strategies and can be achieved in part through xenodiagnosisexperiments. We searched PubMed up to Aug 29, 2019, for English-language articles using the search terms “Leishmaniasis AND xenodiagnosis AND visceral leishmaniasis”. This search yielded 49 papers. An updated search of PubMed combining the original terms with terms (including synonyms and closely associated words) such as “kala-azar”, “post kala-azar dermal leishmaniasis”, and “asymptomatics”, and searching the reference lists of all identified papers, yielded two additional papers. 36 papers reported the results of xenodiagnoses done on animal reservoirs, and 14 studies were done in humans, including one case report. These studies provide sufficient evidence to consider xenodiagnosis the optimal method for identifying the functional reservoir of *L donovani*.

**Added value of this study**

To our knowledge, this study is the first to investigate the infectiousness to sandflies of patients with visceral leishmaniasis or post-kala-azar dermal leishmaniasis following treatment and of people with asymptomatic infection living in areas with high *L donovani* transmission. We found that patients with active visceral leishmaniasis or post-kala-azar dermal leishmaniasis, but not asymptomatic individuals or treated patients, were infectious to vector sandflies. We also found that infectiousness increased with severity of disease in patients with active visceral leishmaniasis. Treatment of patients with visceral leishmaniasis or post-kala-azar dermal leishmaniasis with currently recommended drugs was highly effective in reducing their infectiousness to flies.

**Implications of all the available evidence**

The ongoing strategy to eliminate visceral leishmaniasis from the Indian subcontinent relies on vector control and reducing the number of human infection reservoirs. Our findings, as well as the findings of previous studies, suggest that early case detection followed by complete treatment and localised vector control should suffice to sustain the elimination effort.

Visceral leishmaniasis in the Indian subcontinent is believed to have an anthroponotic transmission cycle as there is no direct evidence that non-human hosts can transmit infection to the sandfly vector.^9^, ^10^ With a decreasing number of active visceral leishmaniasis cases in the community, the relative importance of other infectious groups in the transmission dynamics is increasing.^11^ Both patients with post-kala-azar dermal leishmaniasis, who have skin eruptions as sequelae of treated visceral leishmaniasis, and those with HIV co-infection, who can have repeated clinical visceral leishmaniasis episodes, have been shown to be infectious to sandflies.^12^, ^13^ Furthermore, mathematical modelling posited that people with asymptomatic infection might contribute to transmission, although their infectiousness to sandflies is not yet established.^10^, ^14^ Xenodiagnosis is the most direct way to determine the infectiousness of an infected host to vector sandflies and can be used to investigate the role of potential reservoir populations in the shifting ecology of *L donovani* transmission.^9^ The aim of this study was to use xenodiagnosis to evaluate the relative infectiousness to sandflies of patients with visceral leishmaniasis or post-kala-azar dermal leishmaniasis, before and after treatment, and asymptomatic individuals, so to provide missing information about *L donovani* transmission dynamics for timely incorporation into the visceral leishmaniasis elimination programme in India.

## Methods

### Study design and participants

This study was approved (Dean/2015/CACE/1146 and Dean/2014-15/EC/1159) by the institutional review committees of Banaras Hindu University, Varanasi, India, and Kala-Azar Medical Research Center (KAMRC), Muzaffarpur, India. In this prospective cohort study, we enrolled patients with visceral leishmaniasis, patients with post-kala-azar dermal leishmaniasis, and asymptomatic individuals. Patients with visceral leishmaniasis or post-kala-azar dermal leishmaniasis were recruited when they presented to the KAMRC hospital. Asymptomatic individuals were identified through screening of finger-prick blood samples collected from 17 254 individuals living within 26 high-transmission clusters (in which 167 visceral leishmaniasis cases had been reported over a 3-year period) during two surveys done in 2017–18. Individuals whose blood samples tested positive for anti-leishmania antibodies by direct agglutination test (DAT; Institute of Tropical Medicine, Antwerp, Belgium), rK39 ELISA, or both, in either survey were invited to enrol in the study. Detailed ethics statements, screening procedures, and selection criteria for asymptomatic patients are in the appendix (pp 2–3). We excluded pregnant or lactating women, individuals who had received any vaccination within the past 30 days, and individuals with hepatitis B or C. Eligible participants were HIV negative and aged 12–64 years. Participants provided written informed consent. Illiterate subjects provided a thumb print in the presence of an independent witness who provided a signature. For participants younger than 18 years, written informed consent was obtained from a parent or guardian.

### Procedures

Active visceral leishmaniasis cases were confirmed by a physician at KAMRC hospital on the basis of clinical symptoms (fever for >2 weeks), signs (splenomegaly), positive serology on rK39 rapid diagnostic test, and microscopic demonstration of *Leishmania* spp amastigotes in splenic aspirate smears. Parasite density in splenic aspirate smears was graded microscopically and assigned a splenic score of 1–5 for densities ranging from one to ten amastigotes per 1000 fields to ten to 100 amastigotes per field (appendix p 4). All patients received a single, intravenous dose of liposomal amphotericin B (AmBisome; Gilead Sciences, Foster City, CA, USA; 10 mg/kg) and were followed up for 6 months to assess clinical cure. Nodular and macular post-kala-azar dermal leishmaniasis cases were confirmed by demonstration of parasites in slit-skin smears or by PCR using blood samples. All patients with post-kala-azar dermal leishmaniasis received treatment with 100 mg per day miltefosine (Vardhman Exports, Navi Mumbai, India), given orally for 12 weeks, and were followed up for 6 months to assess clinical cure. All asymptomatic participants were followed up once a month for 24 months after enrolment to monitor for development of active visceral leishmaniasis.

Xenodiagnosis was done on patients with visceral leishmaniasis (before treatment and 30 days after treatment), patients with post-kala-azar dermal leishmaniasis (before treatment and 12 weeks after treatment), and asymptomatic participants at enrolment. For xenodiagnosis, we first established and maintained a laboratory-reared colony of *P argentipes* in an insectary at the KAMRC that was designed and built to meet Arthropod Containment Level 2 (Biosafety Level 2) standards specified by the American Committee on Medical Entomology.^15^ The colony was screened for the absence of sandfly-borne viruses known to occur in India.^15^ We then exposed study participants to 30–35 female and ten to 12 male sandflies for 30 min on each site of the participants' forearms and lower legs, or the forearms only (depending on participant consent). For patients with post-kala-azar dermal leishmaniasis, sandflies were also exposed to nodular and macular lesions. 60–72 h after feeding, we dissected the blood-fed flies. We used microscopic examination of individual sandfly midguts to determine the proportion of sandflies infected and quantitative PCR (qPCR) of midgut homogenates to estimate parasite load. Further details on sandfly-feeding and xenodiagnosis experiments are available in the appendix (p 3)).

In addition to performing DAT-based and rK39 ELISA-based serological tests as the primary measure of asymptomatic infection, we also performed an antigen-specific interferon-γ release assay (IGRA) on whole blood collected from these individuals as a secondary indicator of their sub-clinical exposure. qPCR was also done on whole blood collected from all participants, including people who were asymptomatic, to measure parasite load in blood. Details of DAT, rK39 ELISA, IGRA, splenic parasite scoring, and qPCR performed on blood, skin biopsies, and sandflies are in the appendix (pp 3–5).

### Outcomes

The primary endpoint was the proportion of participants who were infectious to sandflies, with a participant considered infectious to sandflies if one promastigote or more was observed in one sandfly or more by microscopic exam, or if at least one pool of sandflies tested positive by qPCR. The percentage of blood-fed sandflies that were infected and the qPCR values of midgut homogenate were secondary endpoints.

### Statistical analysis

We compared qPCR values, indicating parasite load, for peripheral blood before versus after treatment, for nodular versus macular post-kala-azar dermal leishmaniasis lesions, and for sandflies fed on the forearm versus on the dermal lesions of patients with post-kala-azar dermal leishmaniasis using paired Student's t test in GraphPad Prism, version 7.03. p values less than 0·05 were considered significant.

We estimated Spearman's correlation coefficients, with 95% CIs and p values, to assess the association between splenic scores and blood qPCR values and between blood qPCR values and skin qPCR values using the default cor.test function in R (version 1.1.21) and rank transformations on the values, also in R.^16^ To determine the probability of progression to disease in asymptomatic individuals who were seropositive versus seronegative at baseline, we calculated risk ratios and 95% CIs using logistic regression. To obtain an upper confidence limit (one-sided 97·5% CI) for the proportion of asymptomatic people who are infectious to sandflies, we used exact binomial methods, assuming the fly dissecting assay had a sensitivity of 100% (appendix p 3).

To model the expected percentage of surviving, blood-fed flies that would become infected per person as related to either splenic score or whole-blood qPCR score, we used logistic regression with a random effect for each participant (lme4 R package version 1.1.15) and calculated odds ratios (ORs) with 95% CIs as an effect measure. We assumed that the dissected flies could be treated as a random sample of the surviving, blood-fed flies.

To analyse the relationship between disease severity of participants, defined by parasite load in whole blood, and the number of parasites transmitted to flies, we modelled the log_10_ transformation of the mean sandfly midgut-homogenate qPCR value per pool of sandflies by the log_10_ transformation of the whole-blood qPCR values on day 0 using a mixed-effects weighted linear model, with weights proportional to the number of flies in each pool, and including a random effect for each participant. The mixed-effects models for patients with post-kala-azar dermal leishmaniasis were similar to those for patients with active visceral leishmaniasis, except if needed we allowed separate effects depending on the post-kala-azar dermal leishmaniasis type (macular or nodular) or xenodiagnosis location (forearm or lesion). Mixed-effects models were fit using the lme4 R package (version 1.1.15).

We calculated risk ratios with 95% CIs to test for the association between initial DAT or rK39 seropositivity and clinical visceral leishmaniasis.

Assuming that less than one in 1000 asymptomatic people can infect a sandfly, we estimated that we needed at least 183 participants with asymptomatic infection to show with at least 90% power that the proportion of asymptomatic people who are infectious to sandflies is less than 2%.

### Role of the funding source

The funder of the study had no role in the study design, data collection, data analysis, data interpretation, or writing of the report. All authors had full access to all the data in the study and had final responsibility for the decision to submit for publication.

## Results

Between July 12, 2016, and March 19, 2019, we enrolled 287 participants, including 77 with active visceral leishmaniasis, 26 with post-kala-azar dermal leishmaniasis, and 184 with asymptomatic infection. A timeline of the study from recruitment to completion, as well as a flow diagram of the number of individuals participating in various stages of the study, are provided in appendix p 10. Demographic and clinical data for all enrolled participants are presented in appendix p 6.

**Figure 1:**
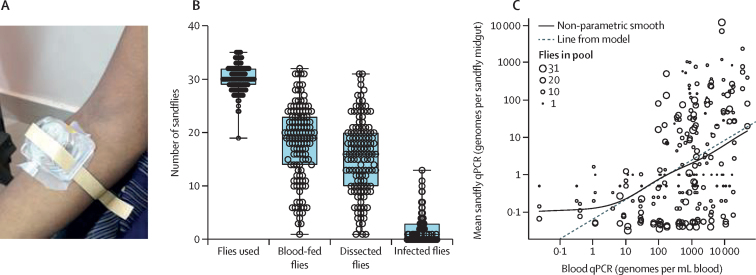
Infectiousness of patients with active visceral leishmaniasis to sandflies (A) Exposure of sandflies in a feeding cup to the forearm during xenodiagnosis. (B) Boxplot of number of exposed, blood-fed, dissected, and infected sandflies in xenodiagnoses. (C) Modelling the mean sandfly qPCR value by the blood qPCR value; each circle represents a pool of sandflies fed on either the forearm or lower leg and either dissected or dead before dissection, such that for each of the 77 patients with visceral leishmaniasis there are one to four circles, accounting for different combinations of feeding site and whether the sandflies were dissected. qPCR=quantitative PCR.

In the 77 patients with parasitologically confirmed active visceral leishmaniasis, a significant association was observed between splenic score and blood qPCR value before treatment (appendix p 11). The median parasite load in these patients before treatment was 844·20 genomes per mL blood (IQR 99–2850), and 71 (92%) patients were successfully treated (<1·0 genome per mL) with a single dose of liposomal amphotericin B (appendix p 11). To assess the relationship between the parasite load in the blood or spleen and the capacity of a patient with visceral leishmaniasis to transmit infection before treatment (table 1), we exposed 4086 female sandflies in xenodiagnosis experiments to 77 patients with visceral leishmaniasis, with a mean of 53 flies (SD 14) exposed to each patient (a mean of 30 flies [SD 2] in the forearm feeding chamber and 23 [SD 2] in the leg feeding chamber; figure 1A–B). 2487 (61%) flies fed during xenodiagnoses, of which 374 (15%) died before dissection and were thus not dissected. 2113 (85%) of 2487 blood-fed flies were dissected and 225 (11%) were found to be infected by microscopy (figure 1B). 42 (55%) of 77 patients with visceral leishmaniasis transmitted parasites to at least one fly.

**Table 1:** Logistic regression analysis for infection in sandflies blood-fed on patients with active visceral leishmaniasis (n=77) grouped according to splenic score and blood qPCR score

	**Number of participants who infected at least one sandfly**	**Expected proportion of flies infected (95% CI)**	**Odds ratio (95% CI)**
**Splenic score**			
1	4/27 (15%)	0·46% (0·14–1·44)	1 (ref)
2	8/14 (57%)	6·57% (2·66–14·87)	15·3 (3·8–61·6)
3	14/19 (74%)	7·74% (3·26–14·78)	18·3 (4·9–67·8)
4	14/15 (93%)	18·87% (9·04–34·88)	50·7 (12·8–200·6)
5	2/2 (100%)	24·07% (3·76–71·92)	69·2 (6·7–710·1)
**qPCR score (parasite genomes per mL of blood)**			
1–10	1/11 (9%)	0·19% (0·02–2·30)	1 (ref)
11–100	1/8 (13%)	0·27% (0·03–2·48)	1·4 (0·1–35·5)
101–1000	15/22 (68%)	5·46% (2·58–11·24)	30·5 (2·4–393·7)
1001–10 000	18/28 (64%)	7·84% (4·03–14·73)	44·9 (3·6–564·4)
>10 000	7/8 (88%)	17·39% (6·22–40·10)	111·3 (7·4–1673·9)

Data are n/N (%) unless otherwise specified. Splenic scores correspond to microscopically determined parasite densities of one to ten amastigotes per 1000 fields (score 1), one to ten amastigotes per 100 fields (score 2), one to ten amastigotes per ten fields (score 3), one to ten amastigotes per field (score 4), and ten to 100 amastigotes per field (score 5; appendix p 4). qPCR=quantitative PCR.

By use of a random-effects model, we found that the probability of infection in flies increased with increasing severity of visceral leishmaniasis disease, both when defined on the basis of splenic score and blood parasitaemia (table 1). Logistic regression estimates using patients with parasite loads of one to ten genomes per mL blood as the reference group showed no significant increase of infection in flies that fed on patients with parasite loads of 11–100 genomes per mL blood, whereas flies that fed on patients with parasite loads of 101 genomes per mL blood or higher had increasingly greater odds of becoming infected (table 1).

We also determined the infectiousness of patients with visceral leishmaniasis to sandflies on the basis of the qPCR values of pooled sandflies. Overall, 60 (78%) of 77 patients with visceral leishmaniasis transmitted infection detectable by qPCR in at least one pool of blood-fed flies. Using a linear mixed model, we found that for each ten-fold increase in blood qPCR value, the mean sandfly qPCR value in the pooled flies increased 3·35 times (95% CI 2·13–5·26; figure 1C). Importantly, all patients who were xenodiagnosis positive by microscopy were also xenodiagnosis positive by qPCR.

Six (8%) of 77 patients with visceral leishmaniasis who were clinically cured with liposomal amphotericin B were still positive on qPCR in peripheral blood, although at values just above the test threshold (1·0–2·5 parasite genomes per mL of blood). None of the sandflies that fed on the cured patients were positive on microscopy; however, seven (9%) of 77 patients were still infectious to flies, as determined by qPCR measured in pools of flies (appendix p 7). Of these seven infectious patients, only one was blood qPCR positive. All of the positive pools had less than 2·27 parasite genomes per sandfly, reflecting transmission of low numbers of parasites and little or no subsequent replication in the fly.

**Figure 2:**
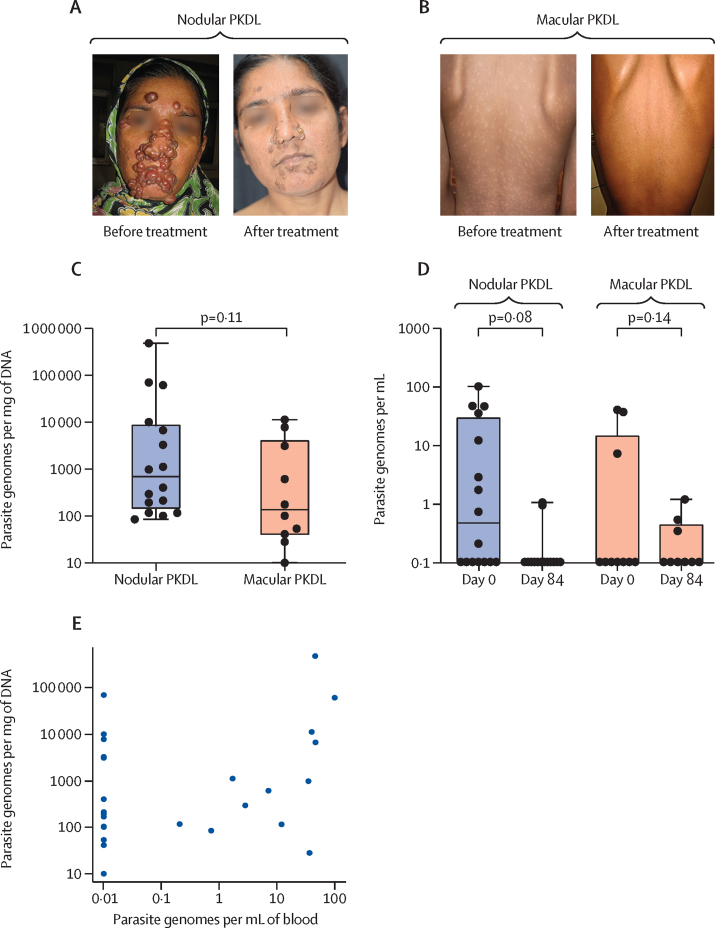
Parasite loads in patients with nodular and macular PKDL (A) Patient with nodular PKDL. (B) Patient with macular PKDL. (C) Boxplots of parasitaemia in lesional tissues from patients with PKDL, as determined by qPCR. (D) Boxplots of parasitaemia in blood of patients with PKDL, before (day 0) and after (day 84) treatment, as determined by qPCR. (E) Scatter plot of blood qPCR values versus skin qPCR values. PKDL=post-kala-azar dermal leishmaniasis. qPCR=quantitative PCR.

26 patients with confirmed post-kala-azar dermal leishmaniasis were enrolled for xenodiagnosis experiments before and after treatment. 16 patients had nodular lesions and ten patients had macular or maculopapular lesions (figure 2A, B; appendix p 6). There was no difference in parasite loads measured by qPCR between tissue from nodular lesions and tissue from macular lesions (figure 2C). Parasite numbers in blood of both types of post-kala-azar dermal leishmaniasis were low compared with parasite numbers in skin lesions or blood from patients with active visceral leishmaniasis, and were reduced to below detectable levels in 21 of 23 patients with post-kala-azar dermal leishmaniasis following miltefosine treatment (figure 2D). There was no significant correlation between blood qPCR and skin qPCR values, although the association was significant among the 12 individuals with detectable blood qPCR (*r*=0·64, 95% CI 0·10–0·89; figure 2E).

**Figure 3:**
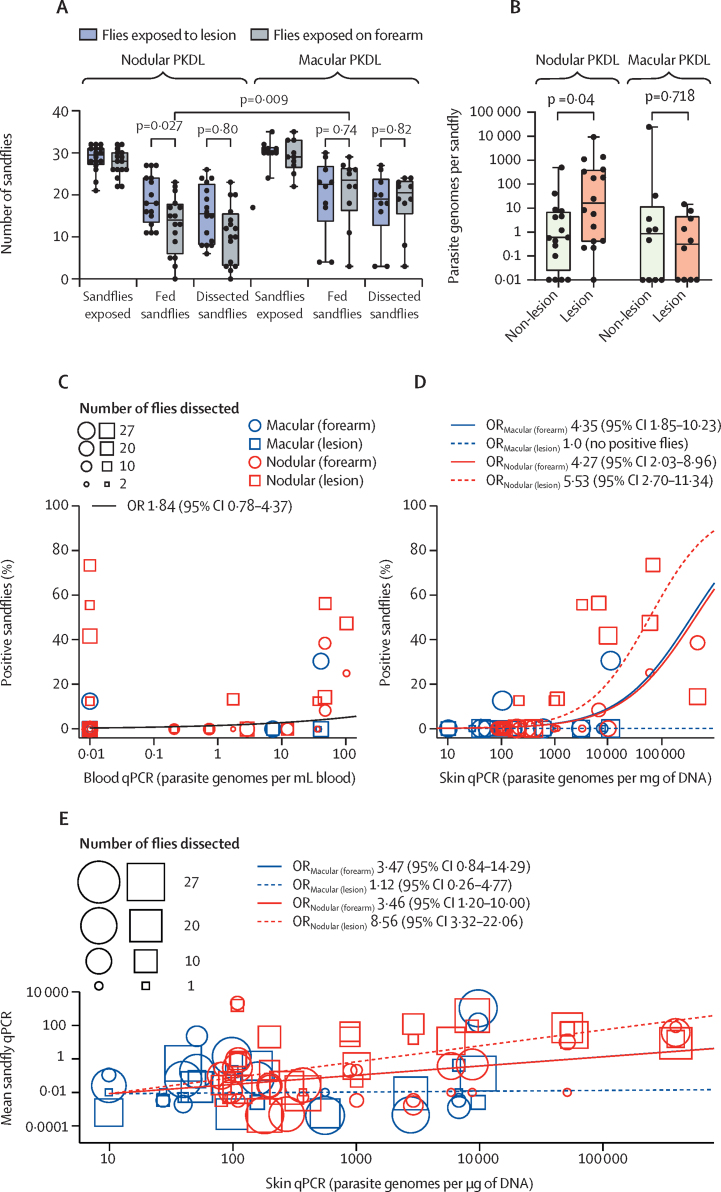
Infectiousness of patients with PKDL to sandflies (A) Boxplots of the numbers of sandflies used, blood-fed, and dissected following feeds on patients with nodular or macular PKDL during xenodiagnosis. (B) Boxplots of parasite loads per sandfly determined by qPCR of the midgut pools from flies fed on the forearm (non-lesion) and lesional sites. (C) Percentage of sandflies infected by blood qPCR value of patients with PKDL; each participant has up to two symbols for flies fed on forearms or lesions. We plotted the predicted response from a typical individual from the mixed-effects logistic regression, with the log_10_-transformed blood qPCR as the only fixed effect and zero values for blood qPCR plotted at 0·01. (D) Percentage of sandflies infected by skin qPCR value of patients with PKDL; each participant has up to two symbols for flies fed on forearms or lesions. Curves are predicted responses from individuals with each combination of xenodiagnosis location and PKDL type from the mixed-effects logistic regression, with the log_10_-transformed skin qPCR as the only fixed effect and with zero values for skin qPCR set to log_10_(10)=1; the macular (lesion) group had no positive flies observed, so we set its effect to zero in the model so that it would converge. ORs show, for each group, the increased odds of infecting a sandfly with every ten-fold increase in skin qPCR. (E) Effect of skin qPCR values on mean qPCR values per fly in sandfly pools; each datapoint represents a pool of sandflies, and each participant has from one to four datapoints. Lines are the model for the four types (the macular [forearm] and nodular [forearm] lines are indistinguishable). ORs show, for each group, the fold increase in the average sandfly qPCR value for each ten-fold increase in skin qPCR. OR=odds ratio. PKDL=post-kala-azar dermal leishmaniasis. qPCR=quantitative PCR.

1495 female sandflies were exposed to the forearms (non-lesion) and to lesions (nodules or macules) of the 26 patients with post-kala-azar dermal leishmaniasis before treatment, with a mean of 30 flies (SD 3) in each feeding chamber. 913 (61%) sandflies took a blood meal (figure 3A, B). Of the 777 blood-fed flies still alive and available for dissection after 62 h, only 68 (9%) were found to be infected by microscopy, with 11 (42%) of 26 patients (nine nodular and two macular) transmitting parasites to at least one fly (appendix p 8). By contrast, 15 (94%) of 16 patients with nodular post-kala-azar dermal leishmaniasis and eight (80%) of ten patients with macular post-kala-azar dermal leishmaniasis transmitted *L donovani* to at least one sandfly as determined by qPCR on pools of sandflies that were fed on forearms or lesions (appendix p 8). In patients with nodular post-kala-azar dermal leishmaniasis, parasite loads as determined by qPCR were significantly higher in the midgut pools from flies that fed on lesions than in the pools of flies that fed on forearms; by contrast, no site difference was observed for the flies fed on patients with macular post-kala-azar dermal leishmaniasis (figure 3B).

The percentage of surviving, blood-fed flies expected to be infected per patient with post-kala-azar dermal leishmaniasis was not affected by the parasite load in blood (OR 1·84, 95% CI 0·78–4·37; figure 3C), whereas it was affected by the skin qPCR value (p<0·0001; figure 3D). We allowed separate effects for each combination of post-kala-azar dermal leishmaniasis type and xenodiagnosis location because they significantly improved the fit of the model compared with random effects (p=0·0005; figure 3D). The model predicts that every ten-fold increase in skin qPCR increases the odds of infecting a fly 1·0–5·53 times, with feeds on nodular lesions showing the highest likelihood of infecting a fly. A linear mixed model on the log scale shows an overall significant effect for skin parasite density in predicting mean sandfly parasite density (p<0·0001), where for each ten-fold increase in skin qPCR, the mean sandfly qPCR in the pooled flies increases 1·12–8·56 times, with the effects significant only for nodular lesions (figure 3E).

Miltefosine treatment of patients with post-kala-azar dermal leishmaniasis resulted in effective parasite clearance, although two patients (one macular and one nodular) were still qPCR-positive for parasites in blood. No follow-up skin biopsies were obtained. 23 (88%) of 26 patients (nine macular and 14 nodular) were available for xenodiagnoses after treatment, of whom only one, the patient with macular post-kala-azar dermal leishmaniasis who was positive on blood qPCR after treatment, remained infectious to flies by qPCR on the pooled flies (appendix p 8). No patient remained positive by microscopy.

184 individuals with asymptomatic infection were enrolled from among the 17 254 individuals who were screened (appendix p 9). Demographic information and serostatus of asymptomatic individuals selected in the first and second serosurveys are summarised in appendix p 12 and table 2. Of the 184 asymptomatic participants, 11 (6%) were qPCR positive in peripheral blood (ie, they had more than one parasite genome per mL) and 83 (45%) tested positive with IGRA (table 2, appendix p 13).

**Table 2:** Serostatus, blood parasitaemia status, and infectiousness to sandflies of 184 participants with asymptomatic infection

	**Enrolled during baseline serosurvey**	**Enrolled during second serosurvey**	**Visceral leishmaniasis cases during follow-up**	**IGRA positive**	**Blood qPCR positive**	**Infectious by microscopy**	**Infectious by qPCR**
Highly seropositive on DAT and strongly positive on rK39 ELISA	46	13	2	33	4	0	0
Highly seropositive on DAT and moderately positive on rK39 ELISA	18	3	0	12	2	0	0
Highly seropositive on DAT and negative on rK39 ELISA	14	15	0	8	2	0	0
Moderately seropositive on DAT and strongly positive on rK39 ELISA	19	31	0	25	3	0	0
Seronegative on DAT and strongly positive on rK39 ELISA	4	5	0	0	0	0	0
Moderately seropositive on DAT and moderately positive on rK39 ELISA	0	16	0	5	0	0	0

On DAT, individuals were considered highly seropositive if they had antibody titres of ≥1:25 600, moderately seropositive if they had antibody titres of ≥1:1600 but <1:25 600, and seronegative if they had antibody titres of <1:1600. On the rK39 ELISA, individuals were considered strongly positive if they had ≥23 PP, moderately positive if they had ≥14 PP but <23 PP, and negative if they had <14 PP. Criteria for inclusion of asymptomatic individuals on the basis of their seropositivity on DAT or rK39 ELISA are described in the appendix (p 2). DAT=direct agglutination test. IGRA=interferon-γ release assay. PP=percentage positivity. qPCR=quantitative PCR.

10 506 female sandflies were exposed to the forearms and legs of 184 asymptomatic individuals, with a mean of 60 flies (SD 3) per participant (30 flies [SD 3] in each feeding chamber). 7164 (68%) sandflies took a blood meal, of which 945 (13%) died before dissection (appendix p 14). None of the 6219 blood-fed, dissected sandflies were found to be positive for infection by microscopy or qPCR (table 2). Analysis of the pools of undissected, dead sandflies by qPCR also did not find any positive infections. Thus, using a one-sided 97·5% CI, we are 97·5% confident that that the true probability of transmission from an asymptomatic individual to a sandfly is less than 1·98%. Over a mean follow-up of 24 months (SD 6·8), we found that two (0·46%) of 431 participants (including those not enrolled for xenodiagnosis experiments) who were seropositive at baseline progressed to visceral leishmaniasis disease compared with two (0·032%) of 6187 participants who were seronegative, resulting in a risk ratio of 14·35 (95% CI 1·04–197·4, p=0·047).

## Discussion

Visceral leishmaniasis has long been considered an anthroponotic disease, but it is not known to what extent different exposure groups serve as reservoir hosts that can promote or sustain *Leishmania* spp transmission within the overall local ecology of the disease. Our xenodiagnosis results suggest that sandfly infections in endemic areas are contributed primarily by active visceral leishmaniasis and post-kala-azar dermal leishmaniasis cases, to a much lesser extent by treated patients, and rarely or not at all by asymptomatic individuals. In patients with active visceral leishmaniasis, infectiousness increased with severity of disease, suggesting that early case detection followed by complete treatment will effectively reduce the transmission of *L donovani* in the community.

The proportion of participants with active visceral leishmaniasis who transmitted infection to at least one sandfly was 55% when assessed by microscopy and 78% when assessed by qPCR of pooled sandfly midguts. All patients who were positive by microscopy were also positive by qPCR. Because the molecular approach did not discriminate between dead and viable organisms, it is possible that the flies that were qPCR positive but microscopy negative acquired dead parasites or had infections that did not result in parasite replication. Furthermore, given we evaluated the presence or absence of *L donovani* only during the earliest stage of parasite growth and development in the vector, we do not know which, if any, of the positive flies would go on to carry mature, transmissible infections. Nonetheless, the findings indicate that the majority of patients with active disease can transmit at least a few organisms, and they are consistent with the conclusion, based on the clustering of cases around households with a history of visceral leishmaniasis, that patients with active disease are the principal reservoirs of *L donovani*.^17^

The number of participants with active visceral leishmaniasis was large enough to stratify them into groups defined by disease severity, either splenic score or qPCR blood parasitaemia, to assess the relationship of these parameters with transmissibility. The groups of active cases with the lowest parasite burdens (splenic score 1 or one to ten parasite genomes per mL blood), who were presumably in the early stage of disease, were poorly infectious to sandflies, with fewer than one in 200 blood-fed flies infected after feeding on these groups. By comparison, increasing parasite load in blood and spleen was associated with increasing probability of transmission to flies, such that patients with the most severe disease transmitted infection to roughly one in five blood-fed flies, consistent with a study from Bangladesh.^18^

Although blood parasitaemia might seem the most probable predictor of transmission to flies, the fact that *L donovani* can colonise the skin has left the relative contributions of blood versus skin an open question. The ability of sandflies to pick up *Leishmania infantum* from infected dogs correlated with parasite loads in both blood and skin,^19^ and the infectiousness of immunodeficient mice infected with *L donovani* was correlated with the distribution of parasites in the skin.^20^ Since skin biopsies were not obtained from patients with active visceral leishmaniasis, we do not know whether parasite numbers in this tissue correlate with positive xenodiagnosis. In the hamster model of visceral leishmaniasis, a strong correlation was found between transmission of *L donovani* to sandflies by direct feeding on the hamsters and artificial feeding on their blood, whereas there was no correlation between direct feeding and feeding on their excised skin.^21^

To our knowledge, the infectiousness of patients with visceral leishmaniasis who have been treated for *L donovani* infection has not previously been addressed. We found that treatment of patients with active visceral leishmaniasis with a single dose of liposomal amphotericin B resulted in low or undetectable levels of parasite in the blood at 30 days and greatly reduced infectiousness of patients to flies. Whereas none of the flies were positive by microscopy, pools of flies that fed on seven of the treated patients were positive by qPCR. The fact that the positive qPCR values were just above the threshold for detection and no parasites were observed by microscopy suggest that the qPCR-positive flies did not contain viable organisms.

Patients with post-kala-azar dermal leishmaniasis can have the condition for years without seeking treatment^22^ and thus could be infectious for a longer time than patients with active visceral leishmaniasis. Cases of persistent post-kala-azar dermal leishmaniasis in West Bengal have been identified as the origin of the resurgence of visceral leishmaniasis in that region.^23^ We found that patients with nodular post-kala-azar dermal leishmaniasis were more infectious to sandflies than patients with macular post-kala-azar dermal leishmaniasis, both in the proportion of flies that were infected and in the numbers of parasites transmitted. Skin qPCR values were most predictive of transmission success when flies were fed directly on nodular lesions, confirming the results of studies done in patients with post-kala-azar dermal leishmaniasis in Bangladesh.^18^, ^24^

The role of asymptomatic infections has so far not been addressed with respect to anthroponotic visceral leishmaniasis in the Indian subcontinent. Incident asymptomatic infections are far more frequent than incident disease, with ratios ranging from 6·1:1·0 to 17·1:1·0 in high-endemic villages of India and Nepal.^25^ Thus, even low infectiousness of asymptomatic individuals could affect visceral leishmaniasis transmission, particularly if their parasitological status remains stable and the number of individuals with asymptomatic infection accumulates over time. One modelling approach predicted that asymptomatic infections contributed to 82% of overall *L donovani* transmission.^26^ In the case of *L donovani,* a challenging aspect of studying asymptomatic infections is that they are not uniformly defined: they can be identified with detectable antibodies, markers of cellular immunity, or the presence of parasite DNA.^14^ We used especially stringent criteria for seropositivity to define the healthy, asymptomatic cohort, in whom prevalent and incident infections were defined by high DAT or rK39 titres. We did not observe a single infection, as assessed by either microscopy or qPCR, in the 6219 sandflies that acquired a blood meal from the 184 asymptomatic participants. This finding is consistent with the results of studies done in Brazil in which *Lutzomyia longipalpis* sandflies did not acquire *L infantum* infections from 137 asymptomatic participants, mainly household members of active visceral leishmaniasis cases, although the serological status of asymptomatic participants was not determined.^27^ In another study, sandflies that fed on four of 19 HIV-negative asymptomatic individuals were positive for *L infantum* by qPCR, although none were positive by microscopy.^28^ Although our negative xenodiagnostic results might have been predicted on the basis of the uniformly low levels of *L donovani* in the blood of asymptomatic participants, the possible contribution of skin-dwelling parasites, detected at a high frequency in microbiopsies from asymptomatic individuals in Ethiopia,^29^ could be addressed only by the direct xenodiagnosis procedure. To the extent that *L donovani* might favour different skin anatomical locations, such as trunk over extremities, a possible limitation of our study is that the flies were exposed only to the leg and the forearm. It is also possible that, had we used a different primary case definition for aymptomatic infection, such as positive blood qPCR, we might have identified some infectious individuals, although none of the 11 qPCR-positive individuals in our asymptomatic cohort were infectious. Among the 184 asymptomatic individuals included in our study, we showed that fewer than 2% were infectious to flies, which we consider a non-important contribution to the maintenance of the *L donovani* transmission cycle. If we consider the asymptomatic group in our study as a random sample of the entire asymptomatic population in Bihar, the estimated probability that an asymptomatic individual can transmit *L donovani* is 0% (95% CI 0–0·0198).

Our data suggest that the visceral leishmaniasis elimination effort could be sustained by early detection and prompt treatment of visceral leishmaniasis and post-kala-azar dermal leishmaniasis cases, and that vector control could be confined to households and areas surrounding the households of active cases.

## Data sharing

Research data and other materials (eg, study protocol, ethical statements, statistical analysis plan, and informed consent form) will be made available to the scientific community immediately following the publication of this manuscript and for up to 5 years. Requests for data should be directed to the corresponding author (SS; drshyamsundar@hotmail.com) and lead investigator (OPS; opbhu07@gmail.com). Requests will be assessed for scientific rigour before being granted, and a data sharing agreement might be required.
