# Supplementary material for: Xenodiagnosis to evaluate the infectiousness of humans to sandflies in an area endemic for visceral leishmaniasis in Bihar, India: a transmission-dynamics study
Source: Lancet Microbe. 2021 Jan;2(1):e23–31. doi: 10.1016/S2666-5247(20)30166-X (PMC7869864; doi:10.1016/S2666-5247(20)30166-X)

THE LANCET
Microbe

Supplementary appendix

This appendix formed part of the original submission and has been peer reviewed. We post it as supplied by the authors.

Supplement to: Singh O P, Tiwary P, Kushwaha A K, et al. Xenodiagnosis to evaluate the infectiousness of humans to sandflies in an area endemic for visceral leishmaniasis in Bihar, India: a transmission-dynamics study. *Lancet Microbe* 2021; **2:** e23–31.

SUPPLEMENTARY APPENDIX

Xenodiagnosis to evaluate the infectiousness of humans to sand flies in an area endemic for visceral leishmaniasis in Bihar, India: a transmission dynamics study

Om Prakash Singh (PhD), Puja Tiwary (PhD), Anurag Kumar Kushwaha (MSc), Shakti Kumar Singh (PhD), Dhiraj Kumar Singh (PhD), Phillip Lawyer (PhD), Edgar Rowton (PhD), Rahul Chaubey (PhD) , Abhishek Kumar Singh (PhD), Tulika Rai (MSc), Michael P. Fay (PhD), Jaya Chakravarty (MD), David Sacks (PhD), Shyam Sundar (MD)

Table of contents

Supplementary Methods ……………………………………………………………………2
Ethics statement…………………………………………………… ……………………..2
Asymptomatic subjects and selection……………………………………………………...2
Xenodiagnosis protocol ……………………………………………………………...........3
Laboratory tests…………………………………………………………………………..3
Direct Agglutination Test (DAT): ……………………………………………….............3
rK39 Enzyme-Linked Immunosorbent Assay……………………………………………..3
Splenic parasite score………………………………………………………………..........4
Real-Time Polymerase Chain Reaction (qPCR) to measure parasite load in blood, skin biopsy and sand fly midguts ………………………………………………………… ….4
Whole blood IFN-γ release assay (IGRA) 5
References…………………………………………………………………………….......5

Supplementary Tables……………………………………………………………………..6
Table S 1: Demographic and clinical information on study participants……………………......6
Table S2: Infectiousness of cured VL patients (day-30 after treatment with single dose Ambisome)……………………………………………………………………………7
Table S3: Infectiousness of PKDL patients to sand flies at pre- and post-treatment………… …8
Table S4: Cluster wise summary of total population, number of subjects participation in Serosurveys and in xenodiagnosis…………………………………………….. …9

Supplementary Figures…………………………………………………………………10
Figure S1: Flow diagram of xenodiagnosis study……………………………………………10
Figure S2. Splenic scores and parasitemias in VL patients…………………………………….11
Figure S3: Study clusters Population pyramid……………………………..…………. …..12
Figure S4: IGRA in asymptomatic subjects……………………………………………......13
Figure S5: Average number of sand flies used, blood fed, and dissected following feeds on asymptomatic subjects during xenodiagnosis………………………………………14

Supplementary Methods

Ethics statement:

This work was conducted with ethical approval (Dean/2015/CACE/1146 dated 27-04-2015, and Dean/2014-15/EC/1159, dated: 21-05-2015) obtained from Institutional Review Committees of Banaras Hindu University, Varanasi, India; and Kala-azar Medical Research Centre (KAMRC), Muzaffarpur, India. Each study patient was informed both orally and in writing (in English and Hindi) about the nature of the study, the anticipated risks and benefits, the discomforts to which the patient will be exposed, and their right to discontinue participation at any time of their own free will. The patients who gave written consent were enrolled in the study. In case of illiterate subjects, a thumb print in addition to signature of an independent witness was obtained. For minors under the age of 18 years, informed consent was obtained from a parent or guardian. Subjects aged <13 were excluded from the study based on recommendation of the project Scientific Advisory Committee (SAC) in order to avoid any risk associated to sand fly exposure and community perception. Subjects who were HIV-positive were also excluded from the study as per guidelines of National AIDS Control Organization (NACO).

Asymptomatic subjects and selection

Asymptomatic subjects were screened from 26 high-transmission clusters (total households =2904) with a total population of 17254 persons (48% female and 52% male), in which 167 VL cases were reported over a 3-year period (2013– 2015) (Figure S1 and Table S4) [Appendix pp 6-9]. We conducted two rounds of house to house surveys during which we collected blood samples on filter paper from all consenting individuals aged 12 years and above. Samples were tested for anti-leishmania antibodies by Direct Agglutination Test (DAT) and rK39 ELISA. In the first sero-survey, subjects positive by both DAT and rK39-ELISA (≥ 1:1600 and ≥ 14 percentage positivity (PP), respectively), and highly seropositive by one or both assays (≥1:25,600 and ≥ 23 PP; see Appendix 1 for how these titers were determined) and who met the other inclusion criteria described above, were invited to KAMRC within 14 days of identification for participation in the xenodiagnosis study. Based on the cutoff criteria for differentiating positive from negative tests, 4.3% (286/6617) subjects were rk39 ELISA positive (cutoff of 14 PP) and 4.6% (305/6617) subjects were DAT positive (cutoff 1:1600). Agreement between DAT and rK39 ELISA was weak with a Kappa of 0.51 (95% CI 0.48 - 0.53). Among the rK39 ELISA positives, 60.4 % (173/286) subjects were moderately positive and 39.5% (113/286) subjects were strongly positive. Similarly, among the DAT positives, 45.9% (140/305) subjects were moderately positive and 54.1% (165/305) subjects were strongly positive by DAT. Only 10.7% (61/573) subjects were strongly positive by both tests. From the first serosurvey, a total of 101 asymptomatic subjects who met the other inclusion criteria of enrolment (not pregnant, not vaccinated within the past 30 days, HIV negative, above 12 years of age) participated in xenodiagnosis.

Subjects who were negative for both serology tests in the first sero-survey were retested in the following year. The seroconverters who tested moderately positive or higher on both of assays were invited to enroll in the study. During the second serosurvey, 5378 samples of finger prick blood were collected, and 499 rK39-ELISA seroconversion and 263 DAT seroconversions were documented. Among the rK39 ELISA seroconverters, 76.6% (382/499) subjects were moderately positive and 23.4% (117/499) subjects were strongly positive. Similarly, among the DAT seroconverters, 70.3% (185/263) subjects were moderately positive and 29.6% (78/263) subjects were strongly positive by DAT. A total of 83 asymptomatic subjects from the second serosurvey who met the other inclusion criteria participated in xenodiagnosis.

A 5 ml sample of heparinized venous blood was collected from all 184 asymptomatic subjects, and whole blood/ serum/ plasma samples were stored in aliquots for qPCR analysis. All asymptomatic subjects were monitored monthly for 24 months after enrollment in the study to observe any development of active VL.

Xenodiagnosis protocol

Sand flies from the 8th to 25th colony generations were used in the direct xenodiagnosis study due to insufficient number of flies in earlier generations to start the study. During xenodiagnosis, 3 -4 days old flies were starved for 12 hrs prior to use. Infectivity of the different subject groups was tested with direct feeding of 30 - 35 females and 10-12 males for 30 min on each site on patients' forearm and lower leg, or the forearm only. For the PKDL patients, sand flies were also exposed on nodular and macular lesions. Flies were loaded into a 2-inch diameter polycarbonate feeding chamber with screen-mesh bottom through which the flies fed, and vented on the top to prevent moisture condensation that might entrap flies inside the chamber. The loaded feeding chamber was strapped to the arm or leg by a Velcro strap. After feeding, the flies were released from the feeding chamber into a polycarbonate holding cage where they were segregated (fed and unfed). Blood-engorged female flies were placed in separate 300-ml paper holding containers and held for at least 60 -72 hrs. in an environmental cabinet at 28°C and 85% humidity. The flies were dissected individually 3-5 days post feeding and infection rates determined by microscopic examination of dissected midguts. After microscopy, midgut material on the slides was transferred to 1.5 ml centrifuge tubes containing PBS. Midgut homogenates from all of the flies fed on the same site were pooled, and parasite load estimates were made by real-time quantitative PCR (qPCR), as described below. Blood fed flies that died prior to the time of dissection were pooled separately and analyzed by qPCR. A subject was considered positive for infectivity to sand flies if an infection was observed in one or more individual flies by microscopic exam, or in one or more of the pools of flies by qPCR analysis. The percent blood fed flies infected and the qPCR values were determined as secondary end points. To get an upper confidence limit on the proportion of asymptomatic subjects that are infective, we use exact binomial methods, assuming the fly dissecting assay has sensitivity of 100%. The 100% sensitivity assumption is a convenience assumption. The fly dissecting assay is meant to determine if there are any viable *Leishmania* parasites in the flies, and there is no other gold standard assay to test it against. The qPCR may detect DNA from non-viable Leishmania, so it is not suitable.

Laboratory tests

Direct Agglutination Test (DAT):

DAT was performed using finger prick blood collected on Whatman filter paper as described elsewhere.[1] Briefly, 100 µl blood filter paper eluate (1:400 dilutions) were serially diluted up to 1:51200 with 50 µl DAT buffer in V- bottom well microtiter plates with one positive and one negative control. Fifty µl of DAT antigen (ITM, Belgium) was then dispensed to every well and plates were incubated overnight at room temperature for agglutination.[2] The DAT results were observed against a white background. Samples with a titer of 1: 1600 or above were considered DAT seropositive. For the purpose of enrollment of asymptomatic subjects in xenodiagnosis DAT seropositives were further subdivided into moderately seropositive (DAT titer ≥ 1:1600 to ≤ 1: 25600) and highly seropositive (DAT titer ≥1:25,600), as only the later were found to be at increased risk of disease.[3]

rK39 Enzyme-Linked Immunosorbent Assay

ELISA against rK39 antigen was performed as described previously. [4-6] Briefly, high binding flat bottom 96-well Nunc ELISA plates (Thermo Fisher Scientific, USA) were coated with rK39

(25ng/well) overnight at 4^0C. Plates were blocked with PBS containing 1% (wt/vol) bovine serum albumin (BSA) (VWR, Life Science, USA) for 2 hrs at room temperature. One hundred µl of eluted blood from 5 mm whatman filter paper was added and incubated for 30 minutes. The wells were washed four times with PBS-Tween (PBS-T) and incubated for 30 minutes with protein G-horseradish peroxidase (1:32000 dilution; Thermo Fisher Scientific, USA) in PBS containing 0.1% BSA and 1.0% Tween-20. Plates were washed four times in PBS-T and incubated with 100 µl tetramethyl benzidine (TMB) / Hydrogen peroxide (H_2O_2) (Bangalore Genei, India) substrate for another five minutes. The reaction was stopped by addition of 1N H_2SO_4 and optical density (OD) measurements were taken at 450nm using a micro-titer plate ELISA reader (Molecular Devices, USA). A positive and negative control (filter paper pooled eluates from VL patients and non-endemic healthy controls, NEHC) were run in each plate, and the positive control was used as a reference to calculate a relative value of positivity of each sample, expressed as percentage positivity (PP). Samples having ≥ 14PP were considered positive. ELISA results were again subdivided into rK39 moderately positive (≥ 14 PP to 23 PP) and rK39 strongly positive (> 23 PP).

Splenic parasite score

Splenic needle aspiration from all VL patients was performed at KAMRC as part of the standard of care for parasitologic diagnosis. A smear was prepared on a slide using 50 microliter (50 ul) of splenic aspirate and stained with Giemsa (Sigma Aldrich, USA). Parasite density (splenic score) was graded microscopically using a conventional log scale of 0 (no amastigotes parasite per 1000 oil immersion fields); 1+ (1 -10 amastigotes per 1,000 fields), 2+ (1 -10 amastigotes per 100 fields); 3+ (1 -10 amastigotes per 10 fields); 4+ (1 -10 amastigotes per fields) and 5+ (10 -100 amastigotes per field).[7]

Real-Time Polymerase Chain Reaction (qPCR) to measure parasite load in blood, skin biopsy and sand fly midguts

Quantification of parasites in whole blood and/or buffy coat, skin biopsy and sand fly midguts was done by Real-time Polymerase Chain Reaction (qPCR), as described elsewhere.[8-10] Briefly, DNA was extracted from 200 µl of heparinized venous blood, skin tissue biopsy and dissected midguts (in pooled) using QIAamp DNA blood and tissue mini kit (QIAGEN, GmbH) and QIAamp DNA Investigator kit (QIAGEN, GmbH), following the manufacturers recommendations. DNA concentration of each sample was measured by NanoDrop ND 2000 (Thermo Fisher Scientific, USA). TaqMan based qPCR on each DNA sample was run in triplicate on an Applied Biosystems (ABI) 7500 Real Time PCR system (Thermo Fisher Scientific, USA) to amplify the region of *L. dovovani* kinetoplast minicircle DNA. Nuclease free water (Thermo Fisher Scientific, USA) as well as blood DNA from NEHC and DNA from uninfected laboratory reared sand fly midguts (pooled) were used as negative controls. Absolute quantification of parasite numbers in the test samples were calculated with the specific set of standard samples (DNA from healthy human blood and uninfected sand fly midguts spiked with serial dilution of cultured *Leishmania* parasites) run in parallel to each set of test samples, as described previously [11]. The average qPCR value for each sand fly pool was calculated as pool qPCR value divided by the number of flies in the pool. qPCR threshold ΔCt value was ≤ 35. Above 35 Ct value was considered as qPCR negative. VL subjects were assigned to 5 groups based on blood qPCR value (parasite genomes / ml of blood): 1 = qPCR 0-10; 2 = qPCR 10-100; 3 = qPCR 100-1000; 4 = qPCR 1000-10000; 5 = qPCR 10000-100000. Six drug treated cured VL patients had peripheral blood qPCR positive with values just above threshold (1.0-2.5 parasite genomes/ml of blood). In case of PKDL, only one patient (macular PKDL) was found to have qPCR value just above threshold.

Whole blood IFN-γ release assay (IGRA)

A whole blood assay for detection of antigen -specific IFN-γ production in-vitro was conducted as described previously.[12,13] Briefly, 3 ml of heparinized whole blood was collected and cultured in the absence of antigen (diluent PBS), or stimulated with *L.donovani* soluble antigen (SLA) (10 µg/ml), or with phytohemagglutinin (PHA) (Sigma-Aldrich, Germany) (5 µg/ml) as a positive control. These tubes were incubated in a humified air atmosphere at 37°C with 5% CO_2 for 20-24 hours. Culture supernatants were collected and stored at -20^{0}C until used for cytokine measurement. Antigen-specific IFN-γ levels in the supernatant were measured using BioLegend ELISA Max Dulex Set kit (Cat# 430104; USA). IFN-γ production in response to SLA stimulation was determined by subtracting background levels measured in the non-stimulated (NIL, PBS) samples. Results were considered positive when the IFN-γ concentration in the antigen wells was >52.28 pg/mL; this cutoff was determined from the mean of IFN-γ concentration of 36 Non endemic healthy controls (NEHC) + 3 standard deviations.

References

1. el Harith A, Kolk AH, Leeuwenburg J, et al. Improvement of a direct agglutination test for field studies of visceral leishmaniasis. *J Clin Microbiol* 1988; **26**(7): 1321-5.
2. Jacquet D, Boelaert M, Seaman J, et al. Comparative evaluation of freeze-dried and liquid antigens in the direct agglutination test for serodiagnosis of visceral leishmaniasis (ITMA-DAT/VL). *Trop Med Int Health* 2006; **11**(12): 1777-84.
3. Hasker E, Malaviya P, Gidwani K, et al. Strong association between serological status and probability of progression to clinical visceral leishmaniasis in prospective cohort studies in India and Nepal. *PLoS Negl Trop Dis* 2014; **8**(1): e2657.
4. Burns JM, Jr., Shreffler WG, Benson DR, Ghalib HW, Badaro R, Reed SG. Molecular characterization of a kinesin-related antigen of Leishmania chagasi that detects specific antibody in African and American visceral leishmaniasis. *Proc Natl Acad Sci U S A* 1993; **90**(2): 775-9.
5. Zijlstra EE, Daifalla NS, Kager PA, et al. rK39 enzyme-linked immunosorbent assay for diagnosis of Leishmania donovani infection. *Clin Diagn Lab Immunol* 1998; **5**(5): 717-20.
6. Khanal B, Rijal S, Ostyn B, et al. Serological markers for leishmania donovani infection in Nepal: Agreement between direct agglutination test and rK39 ELISA. *Trop Med Int Health* 2011; **15**(11): 1390-4.
7. Chulay JD, Bryceson AD. Quantitation of amastigotes of Leishmania donovani in smears of splenic aspirates from patients with visceral leishmaniasis. *Am J Trop Med Hyg* 1983; **32**(3): 475-9.
8. Chakravarty J, Hasker E, Kansal S, et al. Determinants for progression from asymptomatic infection to symptomatic visceral leishmaniasis: A cohort study. *PLoS Negl Trop Dis* 2019; **13**(3): e0007216.
9. Ramesh V, Singh R, Avishek K, et al. Decline in Clinical Efficacy of Oral Miltefosine in Treatment of Post Kala-azar Dermal Leishmaniasis (PKDL) in India. *PLoS Negl Trop Dis* 2015; **9**(10): e0004093.
10. Tiwary P, Kumar D, Mishra M, Singh RP, Rai M, Sundar S. Seasonal variation in the prevalence of sand flies infected with Leishmania donovani. *PLoS One* 2013; **8**(4): e61370.
11. Sudarshan M, Sundar S. Parasite load estimation by qPCR differentiates between asymptomatic and symptomatic infection in Indian visceral leishmaniasis. *Diagn Microbiol Infect Dis* 2014; **80**(1): 40-2.
12. Gidwani K, Jones S, Kumar R, Boelaert M, Sundar S. Interferon-gamma release assay (modified QuantiFERON) as a potential marker of infection for Leishmania donovani, a proof of concept study. *PLoS Negl Trop Dis* 2011; **5**(4): e1042.
13. Singh OP, Gidwani K, Kumar R, et al. Reassessment of immune correlates in human visceral leishmaniasis as defined by cytokine release in whole blood. *Clin Vaccine Immunol* 2012; **19**(6): 961-6.

Supplementary Tables

Table S1: Demographic and clinical information on study participants.

	VL cases		PKDL cases		Asymptomatics
	**Day-0**	**Day-30**	**Day- 0**	**Day-30**	**Asymptomatics**
**N**	77	77	26 [#]	26 [#]	184
**Age (years)**	31.74 ±13.53	31.74 ±13.53	33.6 ± 15.1	33.6 ± 15.1	37.7 ± 16.8
**Sex % (M:F)**	49 : 28	49 : 28	19 : 7	19 : 7	85:99
**Platelets (x10^3 U/L)**	100.6 ± 39.01	190.56 ± 62.84	190.38 ±57.20	288.10 ± 78.61	ND
**WBC (cells/ul)**	3622 ±1733	6550 ±2675	8869 ± 2770	9531 ± 3514	ND
**Haemoglobin (g/dl)**	8.89 ± 1.72	10.12 ± 1.5	13.45 ± 2.1	12.76 ± 1.43	ND
**Lymphocyte (% of WBC)**	46.33 ± 10.89	37.07 ± 9.28	27.34 ± 5.89	24.5 ± 8.62	ND
**Neutrophils (% of WBC)**	49.18± 10.95	57.76 ± 9.1	69.38 ± 5.76	70.4 ± 8.72	ND
**SGOT (IU/mL)**	45.16± 31.68	32.63± 19.95	31.08 ± 11.91	30.09 ± 12.05	ND
**SGPT(IU/mL)**	44.91 ±41.13	40.39 ±26.67	32.0 ± 14.2	33.5 ± 19.27	ND
**Na^+ (mEq/L)**	138.2 ± 1.84	131.5 ± 3.31	133.21 ±1.98	132.6 ±1.72	ND
**K^+ (mEq/L)**	3.92 ± 0.26	3.54 ± 1.08	4.05 ± 0.37	4.24 ± 0.422	ND

Abbreviations: VL= Visceral leishmaniasis; PKDL = Post kala-azar dermal leishmaniasis; WBC = White Blood Cells; SGOT = Serum glutamic oxaloacetic transaminase; SGPT = Serum glutamic pyruvate transaminase.
†Mean value ± SD of aggregated data are shown, ND= not done.
16 Nodular & 10 Macular PKDL subjects

Table S2: Infectiousness of cured VL patients (day-30 after treatment with single dose Ambisome)

**Pre Treatment (Day-0)**		**Post Treatment (Day-30) (n=77)**		
Subject group		Number of subjects with positive blood qPCR	Number of subjects who transmit infection to at least one sand fly (microscopy)	Number of subjects who transmit infection (qPCR on pooled flies)
Splenic Score	1+ (n=27)	1 (3.7%)	0	1 (3.7%)
	2+ (n=14)	2 (14.2%)	0	2 (14.2%)
	3+ (n=19)	0 (0.0%)	0	1 (5.2%)
	4+ - 5+ (n=17)	2 (11.7%)	0	2 (11.7%)
	5+ (n=2)	1 (50.0%)	0	1 (50.0%)
Real Time PCR Score (parasites genome /ml of blood)	1-10 (n=11)	0 (0.0%)	0	0 (0.0%)
	11- 100 (n=8)	0 (0.0%)	0	0 (0.0%)
	101 -1000 (n=22)	3 (13.6%)	0	2 (9.0%)
	1001 – 10000 (n=28)	2 (7.1%)	0	3 (10.7%)
	>10000 (n=8)	1 (12.5%)	0	2 (25.0%)

Table S3: Infectiousness of PKDL patients to sand flies at pre- and post-treatment

Subject ID	Pre-treatment						Post- treatment					
	Xenodiagnosis on Lesion			Xenodiagnosis on Non-lesion (Forearm)			Xenodiagnosis on Lesion			Xenodiagnosis on Non-lesion (Forearm)		
	No of blood fed sand flies dissected	Microscopy result (at least one fly positive)	qPCR result	No of blood fed sand flies dissected	Microscopy result (at least one fly positive)	qPCR result	No of blood fed sand flies dissected	Microscopy result (at least one fly positive)	qPCR result	No of blood fed sand flies dissected	Microscopy result (at least 1 fly positive)	qPCR result
**Nodular PKDL**												
2902	23	-	+	20	-	-	16	-	-	9	-	-
2903	21	+	+	13	+	+	14	-	-	19	-	-
2905	24	-	-	23	-	-	22	-	-	26	-	-
2909	9	+	+	3	-	-	ND	ND	ND	ND	ND	ND
2910	6	-	+	10	-	+	19	-	-	19	-	-
2915	15	+	+	0	-	-	12	-	-	22	-	-
2918	8	+	+	3	-	+	ND	ND	ND	ND	ND	ND
2919	15	+	+	2	-	+	30	-	-	26	-	-
2920	19	+	+	4	+	+	21	-	-	13	-	-
2921	17	-	+	14	-	+	5	-	-	17	-	-
2922	8	+	+	10	-	+	16	-	-	9	-	-
2923	26	-	+	20	-	+	23	-	-	23	-	-
2924	8	-	+	12	-	+	4	-	-	26	-	-
2925	11	-	+	9	-	+	22	-	-	23	-	-
2926	16	+	+	12	+	+	16	-	-	28	-	-
2927	24	+	+	16	-	+	15	-	-	17	-	-
**Macular PKDL**												
2901	18	-	-	24	-	-	13	-	-	13	-	-
2906	18	-	+	18	-	+	ND	ND	ND	ND	ND	ND
2907	20	-	-	24	+	+	8	-	-	13	-	-
2908	23	-	+	22	-	-	18	-	-	20	-	-
2911	3	-	-	3	-	-	31	-	-	30	-	-
2912	3	-	+	8	-	-	22	-	-	20	-	-
2913	27	-	+	22	-	+	19	-	+	18	-	-
2914	20	-	+	19	-	+	25	-	-	12	-	-
2916	26	-	+	23	+	+	23	-	-	15	-	-
2917	10	-	-	19	-	+	9	-	-	7	-	-

Abbreviations: (+): positive; (-): negative; ND: not done; qPCR: quantitative PC

Table S4: Cluster wise summary of total population, number of subjects participation in Serosurveys and in xenodiagnosis

Cluster No	Total house holds (HH)	Total no of Male subjects	Total no of female subjects	Total Population	First Serosurvey		Second Serosurvey	
					Filter paper collected (>12 year old subjects)	No of subjects participated in xenodiagnosis	Filter paper collected (>12 year old subjects)	No of subjects participated in xenodiagnosis
**C01**	130	362	343	705	261	2	0	0
**C02**	105	285	280	565	254	10	270	1
**C02**	276	899	790	1689	663	0	770	18
**C04**	82	235	234	469	231	9	175	2
**C05**	142	412	412	824	335	0	281	4
**C06**	65	201	181	382	159	3	154	1
**C07**	114	324	298	622	258	1	211	3
**C08**	158	501	457	958	325	4	0	0
**C09**	76	231	242	473	215	4	177	4
**C10**	62	205	208	413	183	6	143	2
**C11**	61	172	165	337	130	6	124	4
**C12**	147	447	402	849	410	6	319	6
**C13**	223	717	656	1373	525	0	477	10
**C14**	96	291	252	543	199	0	191	4
**C15**	71	243	217	460	219	6	163	4
**C16**	76	234	213	447	162	3	142	2
**C17**	112	347	303	650	275	2	0	0
**C18**	96	280	260	540	193	0	218	12
**C19**	75	219	198	417	190	7	190	0
**C20**	66	193	179	372	186	4	156	2
**C21**	65	224	198	422	192	7	135	4
**C22**	65	211	199	410	207	6	178	0
**C23**	241	760	661	1421	551	11	411	0
**C24**	89	306	306	612	257	0	341	0
**C25**	122	401	338	739	305	3	152	0
**C26**	89	275	287	562	233	1	0	0

Supplementary Figures

Figure S1: Flow diagram of xenodiagnosis study

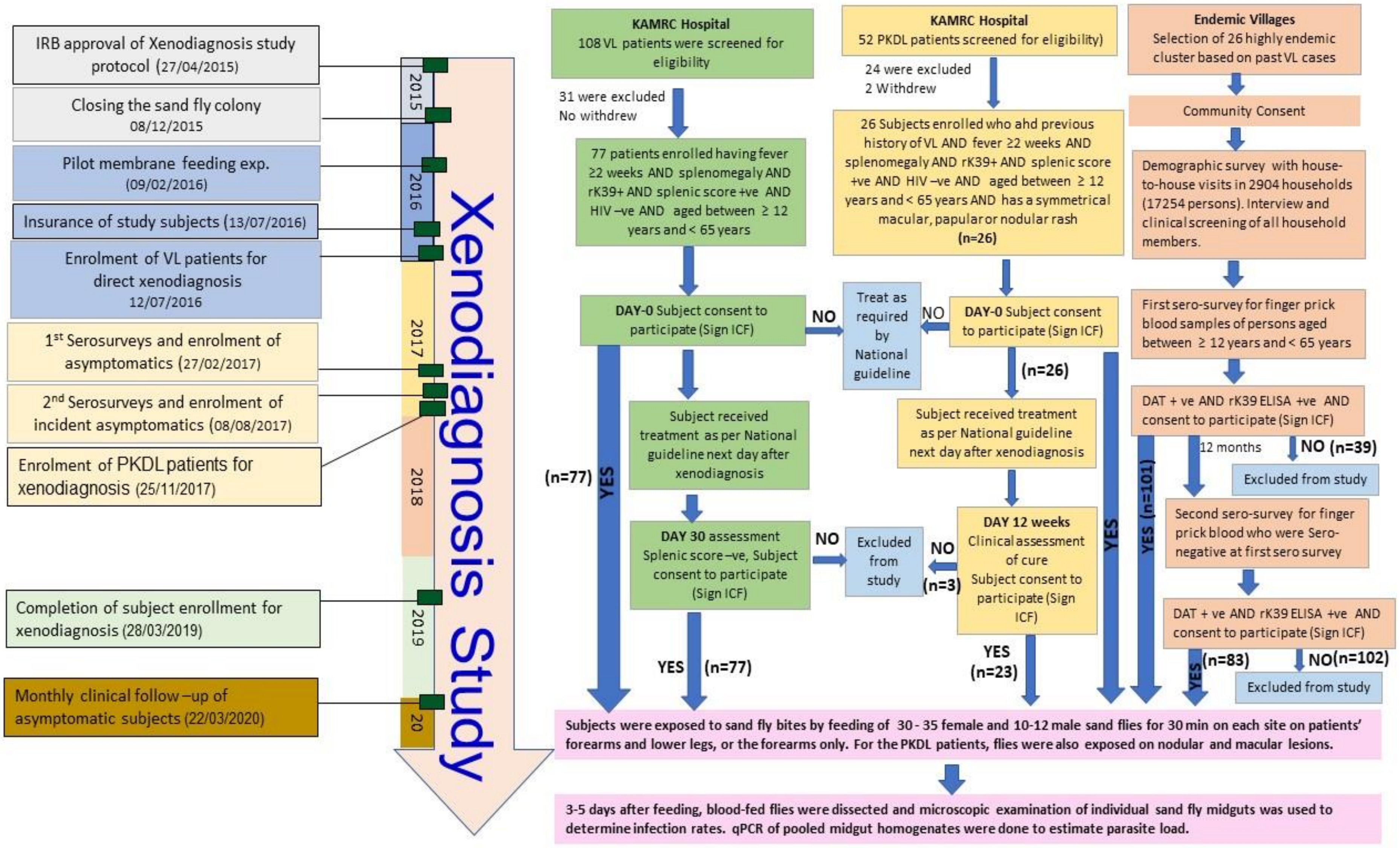

Figure S2: Splenic scores and parasitemias in VL patients. (**A)** Correlation of splenic parasite load with blood parasitemia measured by qPCR; (**B & C)** Parasitemia by qPCR in blood of VL patients before and after treatment; paired samples indicated in **C**. ***p<0.001 determined by paired t test.

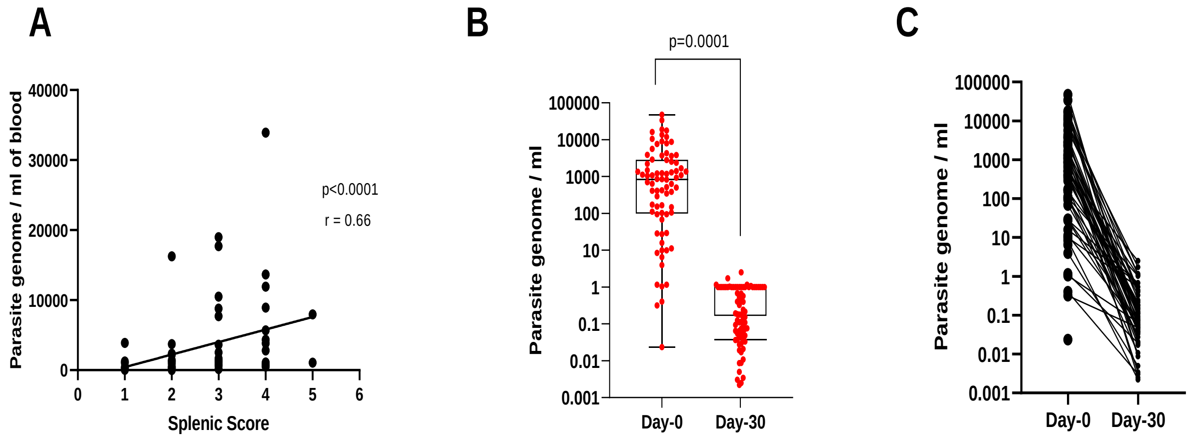

Figure S3: Study Clusters and Population pyramid: (A) Map of study area in Muzaffarpur district showing distribution of study clusters **(B)** population recorded (n=16353) and population participated in finger prick blood Serosurvey.

(A)

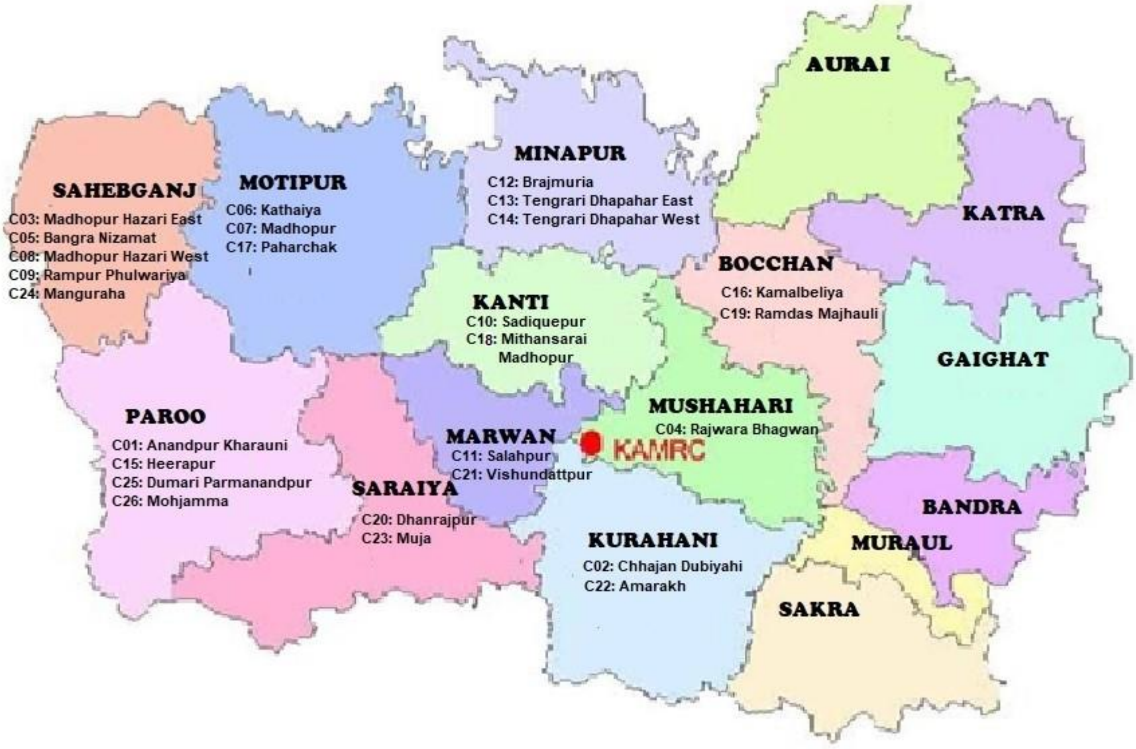

(B)

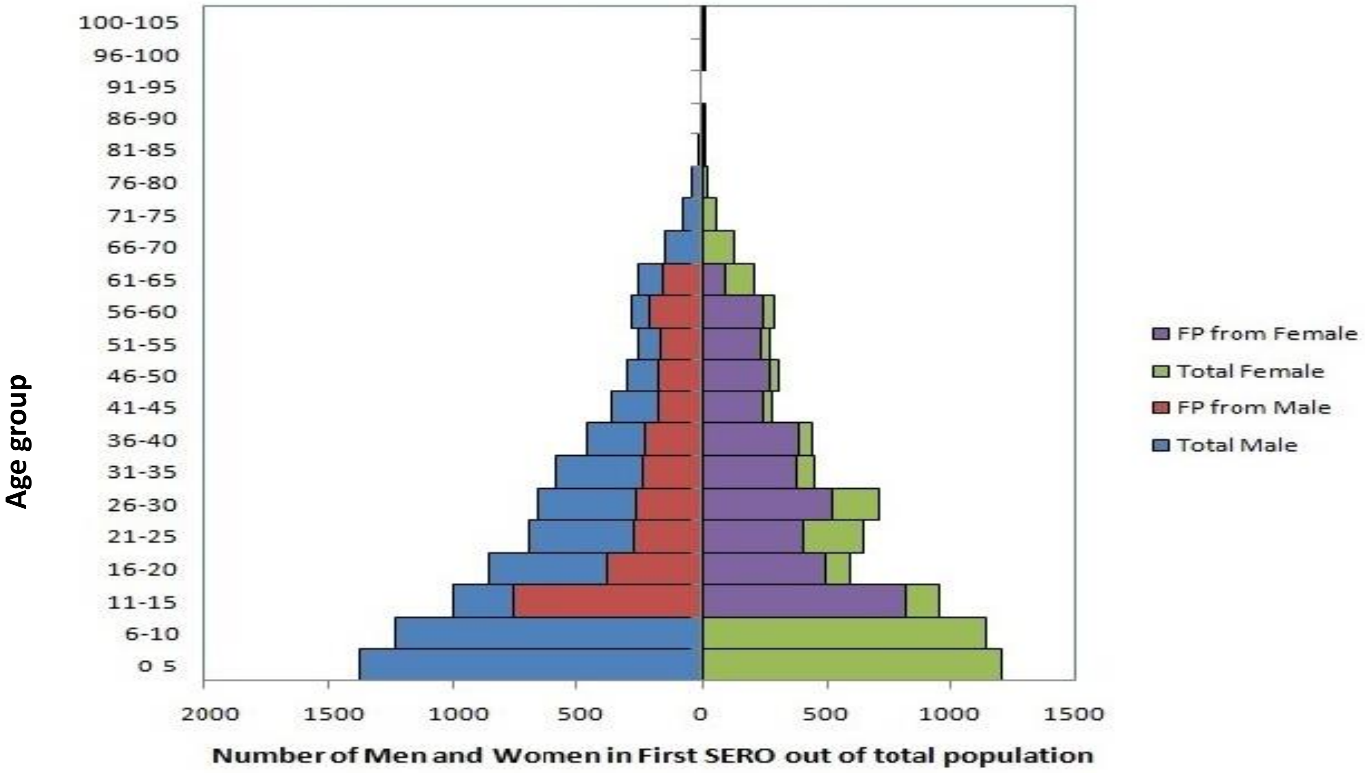

Figure S4: **IGRA in asymptomatic subjects:** (**A)** IFN-γ levels in plasma after stimulation of whole blood cells of asymptomatic subjects with PBS (Nil), SLA and PHA. (**B)** Comparison of SLA-stimulated IFN-γ release (unstimulated NIL value subtracted) for Non endemic healthy controls (NEHC, n=36) and asymptomatic (n=184) subject groups. Bars represent the mean values and the red colour dotted line indicates the cut-off (>52.28 pg/ml) for positivity.

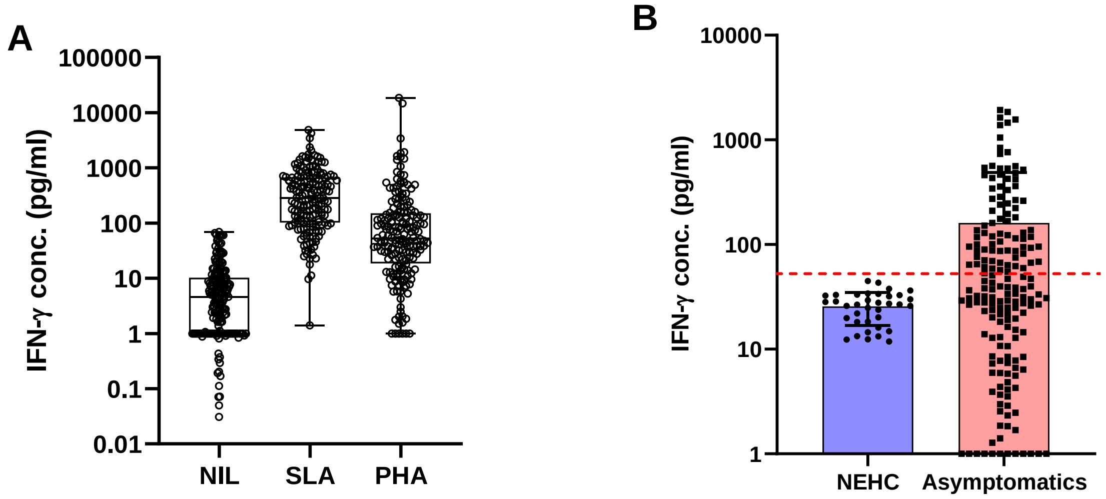

Figure S5: Average number of sand flies used, blood fed, and dissected following feeds on asymptomatic subjects during xenodiagnosis

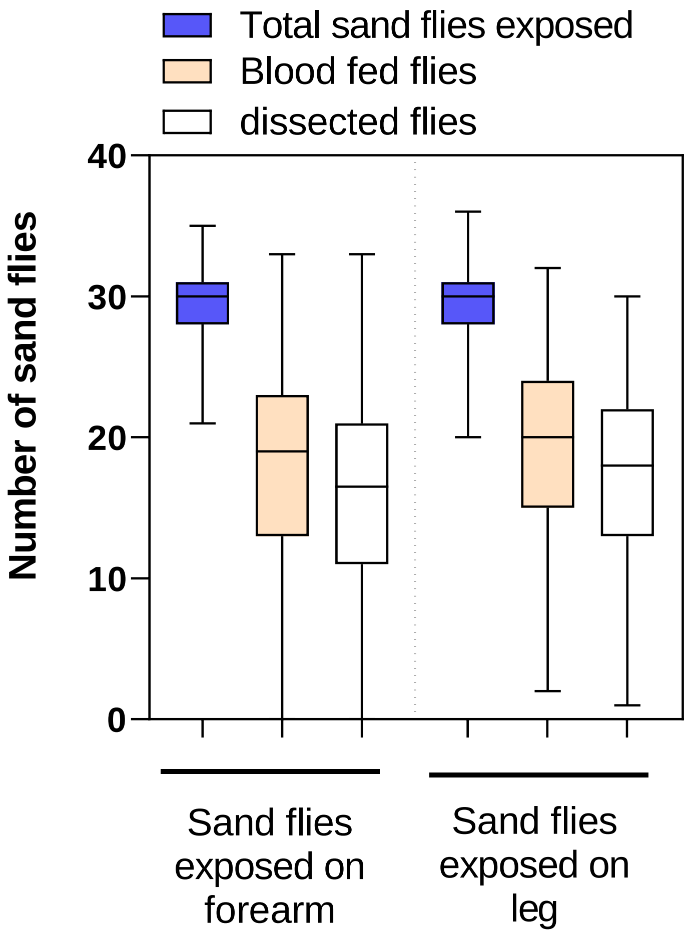

Supplement: Supplementary appendix [file mmc1.pdf]
